# Secondary follicle-like TLS as a protective factor in pMMR rectal cancer: insights into its regional distribution and prognostic value

**DOI:** 10.1093/gastro/goag002

**Published:** 2026-02-13

**Authors:** Jingwen Qi, Han Zhang, Guannan Wang, Zhiqiang Cheng, Tian Liang, Xiangning Huang, Shuhui Huang, Yanan Yin, Xiaoying Lou, Yan Huang

**Affiliations:** Department of Pathology, The Sixth Affiliated Hospital, Sun Yat-sen University, Guangzhou, Guangdong, P. R. China; Guangdong Provincial Key Laboratory of Colorectal and Pelvic Floor Diseases, The Sixth Affiliated Hospital, Sun Yat-sen University, Guangzhou, Guangdong, P. R. China; Biomedical Innovation Center, The Sixth Affiliated Hospital, Sun Yat-sen University, Guangzhou, Guangdong, P. R. China; Department of Pathology, the Affiliated Suzhou Hospital Nanjing Medical University, Suzhou, Jiangsu, P. R. China; Department of Pathology, The Sixth Affiliated Hospital, Sun Yat-sen University, Guangzhou, Guangdong, P. R. China; Guangdong Provincial Key Laboratory of Colorectal and Pelvic Floor Diseases, The Sixth Affiliated Hospital, Sun Yat-sen University, Guangzhou, Guangdong, P. R. China; Biomedical Innovation Center, The Sixth Affiliated Hospital, Sun Yat-sen University, Guangzhou, Guangdong, P. R. China; Department of Pathology, The Sixth Affiliated Hospital, Sun Yat-sen University, Guangzhou, Guangdong, P. R. China; Guangdong Provincial Key Laboratory of Colorectal and Pelvic Floor Diseases, The Sixth Affiliated Hospital, Sun Yat-sen University, Guangzhou, Guangdong, P. R. China; Biomedical Innovation Center, The Sixth Affiliated Hospital, Sun Yat-sen University, Guangzhou, Guangdong, P. R. China; School of Public Health, Capital Medical University, Beijing, P. R. China; Department of Pathology, The Sixth Affiliated Hospital, Sun Yat-sen University, Guangzhou, Guangdong, P. R. China; Guangdong Provincial Key Laboratory of Colorectal and Pelvic Floor Diseases, The Sixth Affiliated Hospital, Sun Yat-sen University, Guangzhou, Guangdong, P. R. China; Biomedical Innovation Center, The Sixth Affiliated Hospital, Sun Yat-sen University, Guangzhou, Guangdong, P. R. China; Department of Pathology, The Sixth Affiliated Hospital, Sun Yat-sen University, Guangzhou, Guangdong, P. R. China; Guangdong Provincial Key Laboratory of Colorectal and Pelvic Floor Diseases, The Sixth Affiliated Hospital, Sun Yat-sen University, Guangzhou, Guangdong, P. R. China; Biomedical Innovation Center, The Sixth Affiliated Hospital, Sun Yat-sen University, Guangzhou, Guangdong, P. R. China; The Affiliated Brain Hospital, Guangzhou Medical University, Guangzhou, Guangdong, P. R. China; Key Laboratory of Neurogenetics and Channelopathies of Guangdong Province and the Ministry of Education of China, Guangzhou Medical University, Guangzhou, Guangdong, P. R. China; Department of Pathology, The Sixth Affiliated Hospital, Sun Yat-sen University, Guangzhou, Guangdong, P. R. China; Guangdong Provincial Key Laboratory of Colorectal and Pelvic Floor Diseases, The Sixth Affiliated Hospital, Sun Yat-sen University, Guangzhou, Guangdong, P. R. China; Biomedical Innovation Center, The Sixth Affiliated Hospital, Sun Yat-sen University, Guangzhou, Guangdong, P. R. China; Department of Pathology, The Sixth Affiliated Hospital, Sun Yat-sen University, Guangzhou, Guangdong, P. R. China; Guangdong Provincial Key Laboratory of Colorectal and Pelvic Floor Diseases, The Sixth Affiliated Hospital, Sun Yat-sen University, Guangzhou, Guangdong, P. R. China; Biomedical Innovation Center, The Sixth Affiliated Hospital, Sun Yat-sen University, Guangzhou, Guangdong, P. R. China

**Keywords:** mismatch repair–proficient rectal cancer, tertiary lymphoid structures, tumor immune microenvironment

## Abstract

**Background:**

Mismatch repair–proficient rectal cancer (pMMR RC) is characterized by limited effective therapeutic options and an unfavorable prognosis, largely attributed to the complexity of the tumor immune microenvironment. Tertiary lymphoid structures (TLSs), which are ectopic lymphoid aggregates that develop within tumors, constitute a crucial component of this microenvironment and have shown considerable prognostic significance. However, the maturation heterogeneity of TLS subtypes within the tumor immune microenvironment, as well as their associations with clinicopathological characteristics and patient outcomes, remain poorly defined. This study aimed to comprehensively characterize the spatial distribution patterns and prognostic relevance of TLS subtypes in patients with pMMR RC.

**Methods:**

We retrospectively analysed tissue sections from 155 patients with pMMR RC who underwent radical resection. TLS maturation subtypes were identified by using hematoxylin and eosin staining and immunohistochemistry, with their spatial distribution quantified and associated with prognosis.

**Results:**

The number of all TLS maturation subtypes in the invasive margin was significantly higher than that in the central tumor region (invasive margin vs central tumor region, *P *< 0.001). Among these, primary follicle-like TLS exhibited the greatest abundance across all tumor regions. Univariate survival analysis revealed that a higher infiltration of secondary follicle-like TLS across all tumor regions was significantly associated with improved survival, whereas increased early TLS infiltration was associated with poorer outcomes. Multivariate analysis further confirmed that the overall infiltration level of secondary follicle-like TLS was an independent predictor of both overall survival and disease-free survival in patients with pMMR RC.

**Conclusion:**

Secondary follicle-like TLS infiltration independently predicts survival in pMMR RC, underscoring its potential as a prognostic biomarker and immunotherapeutic target.

## Introduction

Rectal cancer (RC) remains a leading cause of cancer-related mortality worldwide, with its incidence and mortality rates demonstrating a persistent upward trend [1]. Although significant advances have been achieved with standard treatments such as surgery, radiotherapy, and chemotherapy, patient outcomes in RC remain suboptimal [1–3], emphasizing the pressing need for innovative and more efficacious therapeutic strategies.

In recent years, immune checkpoint blockade (ICB) therapy has achieved remarkable clinical success across several solid tumors, including RC. However, the majority of RC cases are mismatch repair–proficient (pMMR), which exhibit limited responsiveness to ICB and poor clinical outcomes. Increasing evidence suggests that this therapeutic resistance may partly result from insufficient immune cell infiltration into the tumor microenvironment [3]. Tertiary lymphoid structures (TLSs)—ectopic lymphoid aggregates formed in non-lymphoid tissues under chronic inflammatory stimuli (e.g. autoimmune disorders, persistent infections, and malignancies)—represent a lymphangiogenic process driven by sustained exposure to chemokine- and cytokine-mediated inflammatory signals [4, 5]. As a critical component of the tumor microenvironment, TLSs have been strongly linked to antitumor immune responses, immunotherapy efficacy, and patient outcomes [6–8]. Notably, emerging evidence suggests significant TLS compositional differences between responders and nonresponders to ICB [8], suggesting their potential as predictive biomarkers for treatment response. Furthermore, TLSs exhibit heterogeneous maturation states within tumors and their maturation gradient has emerged as a critical determinant of patient prognosis [6]. This association has been validated in several malignancies, including melanoma, clear cell renal carcinoma, bladder cancer, and lung squamous cell carcinoma [9–14]. Nevertheless, the spatial organization and maturation heterogeneity of TLS within the pMMR RC immune microenvironment and their clinical relevance remain poorly characterized.

To address this gap, we systematically characterized the spatial distribution and maturation states of TLSs in the pMMR RC microenvironment by using immunohistochemistry and evaluated their associations with clinicopathological features and prognosis. These findings may provide new insights into the tumor immune architecture and identify potential immunomodulatory targets for optimizing therapeutic strategies in pMMR RC.

## Materials and methods

### Patients and database

In this study, clinicopathological data and paraffin-embedded specimens were collected from 155 patients with pathologically confirmed pMMR RC who underwent radical surgical resection at the Sixth Affiliated Hospital of Sun Yat-sen University (Guangzhou, China) between January 2016 and December 2018. All patients were free of distant metastasis and had not previously received radiotherapy, targeted therapy, or immunotherapy. Clinical and pathological parameters were extracted from medical and pathology records. Pathological diagnosis was based on the World Health Organization (WHO) Classification of Tumors of the Digestive System, 5th Edition [15]. The tumor stage was classified according to the American Joint Committee on Cancer/International Union Against Cancer (AJCC/UICC) Staging Manual (8th Edition) [16]. Clinical and pathological data included age, tumor size, gender, lymphovascular invasion (LVI), perineural invasion (PNI), tumor differentiation, tumor budding, adjuvant chemotherapy, sphincter-preserving, pathological tumor stage (pT), pathological node stage (pN), and pathological tumor-node-metastasis (pTNM) staging system (Table 1).

**Table 1.** Clinicopathological characteristics of 155 patients with pMMR RC

Clinicopathological parameter		*n* (%)
Gender	Male	94 (60.6)
	Female	61 (39.4)
Age (years)	≤61	82 (52.9)
	>61	73 (47.1)
Tumor size^a^ (cm)	≤3.5	95 (61.3)
	>3.5	60 (38.7)
LVI^a^	Negative	136 (87.7)
	Positive	19 (12.3)
PNI^a^	Negative	141 (91.0)
	Positive	14 (9.0)
Tumor differentiation^a^	Poor/moderate	135 (87.1)
	Well	20 (12.9)
pT^a^	pT1–2	27 (17.4)
	pT3–4	128 (82.6)
pN^a^	pN0	84 (54.2)
	pN+	71 (45.8)
pTNM^a^	I	21 (13.5)
	II	63 (40.6)
	III	71 (45.8)
TB^a^	Bd1	56 (36.1)
	Bd2	53 (34.2)
	Bd3	46 (29.7)
Adjuvant chemotherapy	No	71 (45.8)
	Yes	84 (54.2)
Sphincter-preserving	No	22 (14.2)
	Yes	133 (85.8)

a Based on the pathological diagnose.

### Immunohistochemical staining

Formalin-fixed, paraffin-embedded tissue blocks were sectioned into 4-μm–thick slices and mounted onto silane-coated glass slides, deparaffinized in xylene, and rehydrated through graded ethanol (100%, 95%, 90%, 80%, and 70%; 2 min each). Antigen retrieval was performed in citrate buffer (pH 6.0) for 8 min, followed by 3% hydrogen peroxide for 20 min to quench endogenous peroxidase activity and serum-free protein blocking for 30 min. Subsequently, the sections were incubated with primary antibodies at 4°C overnight, followed by incubation with horseradish peroxidase-conjugated secondary antibodies at room temperature for 1 h. The following primary antibodies (Zhongshan Goldenbridge Biotechnology, China) were used: anti-CD3 (#ZA-0503), anti-CD19 (#ZM-0038), anti-CD21 (#ZA-0525), and anti-Bcl6 (#ZM-0011). 3,3′-Diaminobenzidine was used for visualization, producing brown chromogenic signals. Slides were counterstained with hematoxylin, dehydrated, and mounted. All stained slides were scanned and digitized by using a high-resolution scanner (Shengqiang SQS-120P, Shenzhen, China).

### Definition of tumor region division

The number of TLS phenotypes was quantified in distinct anatomical regions according to the standardized protocol of the International Tumor-infiltrating Lymphocytes Working Group [17]. The tumor area was systematically divided into two histological compartments—the central tumor (CT) and the invasive margin (IM)—following established guidelines (**Supplementary Figure S1**).

### TLS definition and detection

Three TLS maturation phenotypes were identified: early TLSs (E-TLSs), primary follicle-like TLSs (PFL-TLSs), and secondary follicle-like TLSs (SFL-TLSs) (**Supplementary Figure S2**) [18, 19]. TLS detection was performed in three steps. First, all lymphocyte aggregates within the tumor tissue were identified. Immunohistochemical staining for CD19 and CD3 distinguished E-TLSs from nonspecific lymphocyte cell clusters. TLSs were excluded if only CD19^+^ or CD3^+^ cell clusters were present without colocalization. The round-shaped cell clusters helped differentiate E-TLSs from PFL-TLSs/SFL-TLSs. Subsequently, Bcl6 and CD21 staining identified mature germinal centers characteristic of SFL-TLSs, thereby distinguishing them from PFL-TLSs. Following this comprehensive morphological and immunohistochemical characterization, the number of TLSs per tissue section was manually quantified by two independent pathologists. Bcl6, a key regulator of B-cell maturation [20], and CD21, a marker of plasma-cell differentiation in germinal centers [21], served as defining indicators for TLS classification.

### Identification and quantification of tumor budding

Tumor budding (TB) was defined as the presence of a single tumor cell or a cluster of up to four tumor cells at the IM of the colorectal cancer. Quantification followed the 2016 International Tumor Budding Consensus Conference recommendations [22]. One “hotspot” field (0.785 mm^2^) at the invasive front was selected and TB was graded as low (Bd1, 0–4 buds/0.785 mm^2^), intermediate (Bd2, 5–9 buds/0.785 mm^2^), and high (Bd3, ≥10 buds/0.785 mm^2^).

### Statistical analysis

Clinicopathological characteristics were systematically recorded and analysed. For analytical purposes, the continuous variable of age was dichotomized at the median. Categorical variables are expressed as frequencies or percentages and were compared by using Pearson’s chi-squared or Yates continuity-corrected chi-squared tests. Data normality was assessed through the Shapiro–Wilk test; Mann–Whitney *U* tests were applied for non-normally distributed continuous variables. Patients were stratified into TLS-negative and TLS-positive groups. Kaplan–Meier survival analysis was used to assess survival, with log-rank tests for univariate comparisons. Variables with *P *< 0.05 were entered into Cox proportional hazards models for multivariate analysis to identify independent prognostic factors. Statistical analysis and graphical representations were performed by using GraphPad Prism (Version 8.0) and IBM SPSS software (Version 22.0). Two-tailed statistical significance was set at *P *< 0.05.

## Results

### Patient characteristics

This study included 155 patients with pMMR RC. Among them, 94 (60.6%) were male, 73 (47.1%) were >61 years old, and 60 (38.7%) had primary tumors >3.5 cm in size. All patients were pathologically diagnosed with adenocarcinoma, including 13 (8.4%) poorly differentiated, 20 (12.9%) well-differentiated, and 122 (78.7%) moderately differentiated cases. LVI and PNI were observed in 19 (12.3%) and 14 (9.0%) patients, respectively. Regarding tumor stage, 27 patients (17.4%) were classified as pT1–2 and 128 (82.6%) as pT3–4. Lymph node metastasis was detected in 71 patients (45.8%). According to the AJCC 8th edition, 21 (13.5%) were stage I, 63 (40.6%) were stage II, and 71 (45.8%) were stage III. For TB assessment, 109 patients (70.3%) exhibited low to intermediate grade (Bd1–2), whereas 46 (29.7%) demonstrated high grade (Bd3). Adjuvant chemotherapy was administered to 84 patients (54.2%), while 71 (45.8%) did not receive postoperative chemotherapy. Sphincter-preserving surgery was successfully performed in 133 patients (85.8%) (Table 1).

### The unique spatial distribution characteristics of TLSs in pMMR RC

**Figure 1.**
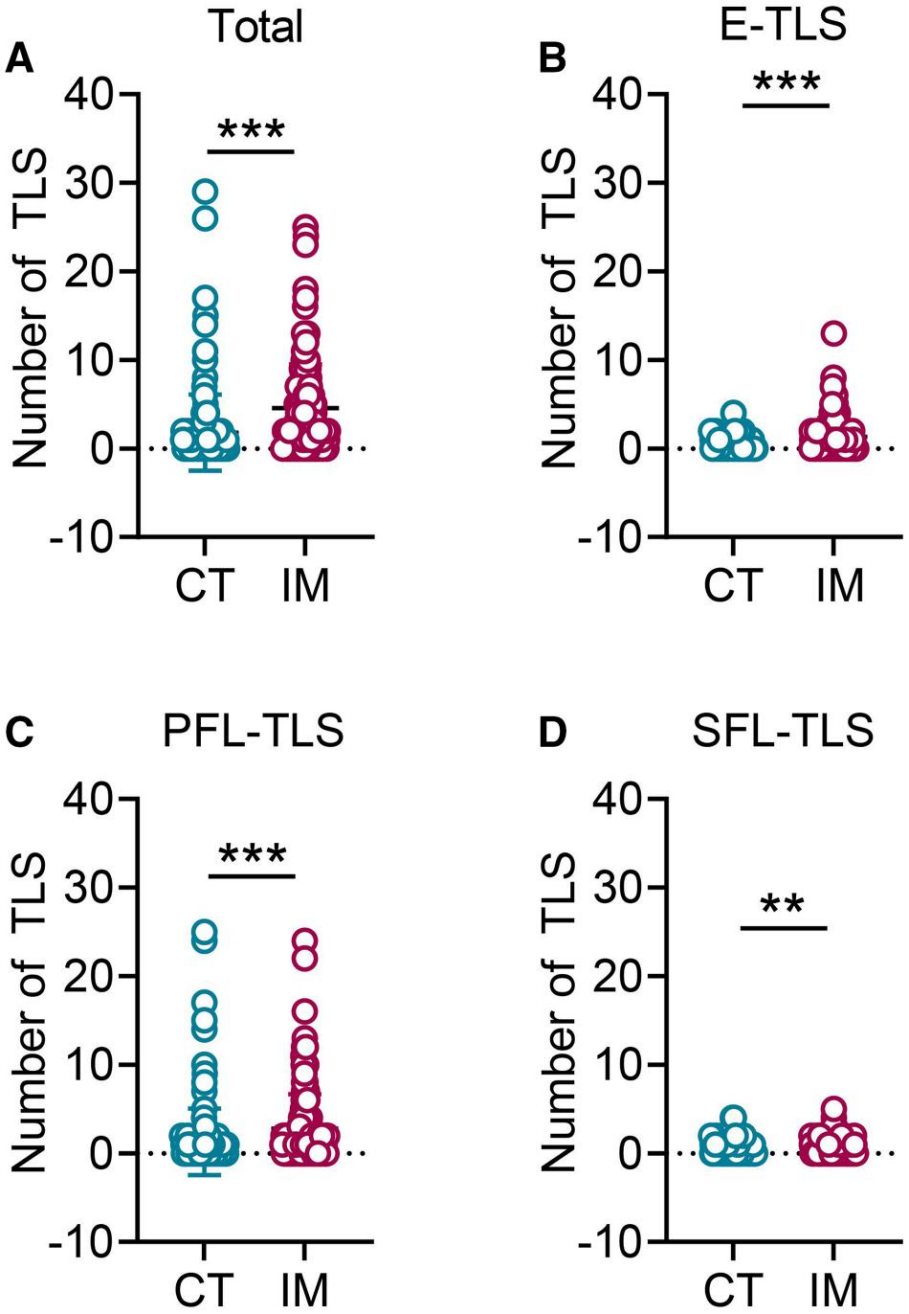
Quantitative distribution of TLSs in subregions of pMMR RC. The number of (A) total TLSs, (B) E-TLSs, (C) PFL-TLSs, and (D) SFL-TLSs in CT and IM. Data are represented as mean ± standard error of the mean. **P < 0.01, ***P < 0.001.

**Figure 2.**
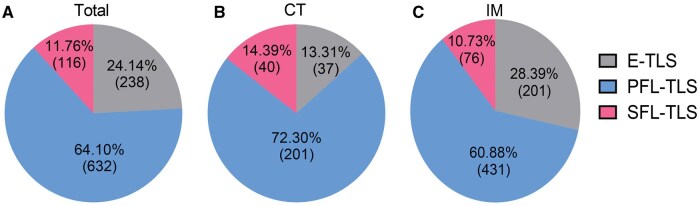
Characteristics of TLS maturation subtype spatial distribution in pMMR RC. Pie charts depict the distribution and proportions of TLS subtypes at different maturation stages in various tumor regions. The distribution and proportions of E-TLSs, PFL-TLSs, and SFL-TLSs in (A) total tumor region, (B) CT, and (C) IM.

A significantly greater number of total TLSs were observed in the IM region than in the CT region (*P ***<** 0.001) (Figure 1A). To determine whether this spatial bias applied to different maturation subtypes, we conducted comprehensive immunohistochemical analyses. Consistently, the numbers of E-TLSs, PFL-TLSs, and SFL-TLSs were all significantly higher in the IM region than in the CT region (*P ***<** 0.001, **<**0.001, and 0.007, respectively) (Figure 1B–D). Further analysis revealed heterogeneous TLS distributions across tumor subregions, which may influence the immune landscape and clinical outcomes [23]. Among all TLS maturation subtypes, PFL-TLSs were the most abundant across all tumor regions (Figure 2).

Our study demonstrated that the TLS maturation subtype spatial distribution in the pMMR RC immune microenvironment followed a consistent pattern, with all subtypes predominantly localize to the IM region and PFL-TLSs being the most prevalent in pMMR RC.

### Association between TLS maturation subtypes and clinicopathological factors

As shown in Figure 1, the TLS spatial distribution in pMMR RC exhibited a consistent pattern, predominantly localized to the tumor IM. Current research indicates that TLSs in the tumor immune microenvironment crucially influence the immune responses of the tumor and the efficacy of immunotherapy [24]. Prior research has associated TLS density with tumor stage, lymph node metastasis, and tumor size [25]. However, the relationship between TLS maturation subtypes and clinicopathological factors in pMMR RC remain unclear. We therefore assessed the associations between the presence of E-TLSs, PFL-TLSs, and SFL-TLSs and clinicopathological features.

Our investigation revealed significant maturation-specific associations with particular clinicopathological indicators. Both the E-TLS–positive and PFL-TLS–positive groups were positively associated with PNI (Table 2). Additionally, the E-TLS–positive group was also positively associated with LVI, the pT stage, and the pTNM stage (Table 2). In contrast, the SFL-TLS–positive group was negatively associated with the pT and pTNM stages (Table 2). Notably, while these significant associations were observed with key pathological progression markers, other baseline characteristics, including patient age, gender, tumor size, and TB, showed no substantial relationships with the TLS maturation patterns, reinforcing the specific connection between TLS maturation states and disease-progression parameters.

**Table 2.** Associations between the status of TLSs with different maturation stages and clinicopathological features in pMMR RC patients (*n *= 155)

Variable	E-TLS_Total_			PFL-TLS_Total_			SFL-TLS_Total_		
	Negative	Positive	***P* value** ^a^	Negative	Positive	***P* value** ^a^	Negative	Positive	***P* value** ^a^
**Gender**			0.660			0.350			0.546
Female	34 (55.7)	27 (44.3)		14 (23.0)	47 (77.0)		38 (62.3)	23 (37.7)	
Male	49 (52.1)	45 (47.9)		28 (29.8)	66 (70.2)		63 (67.0)	31 (33.0)	
**Age (years)**			0.769			0.519			0.411
≤61	43 (52.4)	39 (47.6)		24 (29.3)	58 (70.7)		51 (62.2)	31 (37.8)	
>61	40 (54.8)	33 (45.2)		18 (24.7)	55 (75.3)		50 (68.5)	23 (31.5)	
**Tumor size (cm)**			0.301			0.402			0.315
≤3.5	54 (56.8)	41 (43.2)		28 (29.5)	67 (70.5)		59 (62.1)	36 (37.9)	
>3.5	29 (48.3)	31 (51.7)		14 (23.3)	46 (76.7)		42 (70.0)	18 (30.0)	
**LVI**			0.040			0.236			0.405
Negative	77 (56.6)	59 (43.4)		39 (28.7)	97 (71.3)		87 (64.0)	49 (36.0)	
Positive	6 (31.6)	13 (68.4)		3 (15.8)	16 (84.2)		14 (73.7)	5 (26.3)	
**PNI**			0.012			0.038			0.418
Negative	80 (56.7)	61 (43.3)		42 (29.8)	99 (70.2)		90 (63.8)	51 (36.2)	
Positive	3 (21.4)	11 (78.6)		0 (0)	14 (100)		11 (78.6)	3 (21.4)	
**Tumor differentiation**			0.787			0.378			0.352
Poor	6 (46.2)	7 (53.8)		3 (23.1)	10 (76.9)		7 (53.8)	6 (46.2)	
Moderate	67 (54.9)	55 (45.1)		36 (29.5)	86 (70.5)		83 (68.0)	39 (32.0)	
Well	10 (50.0)	10 (50.0)		3 (15.0)	17 (85.0)		11 (55.0)	9 (45.0)	
**pT**			0.005			0.201			<0.001
pT1–2	21 (77.8)	6 (22.2)		10 (37.0)	17 (63.0)		9 (33.3)	18 (66.7)	
pT3–4	62 (48.4)	66 (51.6)		32 (25.0)	96 (75.0)		92 (71.9)	36 (28.1)	
**pN**			0.514			0.931			0.557
pN0	47 (56.0)	37 (44.0)		23 (27.4)	61 (72.6)		53 (63.1)	31 (36.9)	
pN+	36 (50.7)	35 (49.3)		19 (26.8)	52 (73.2)		48 (67.6)	23 (32.4)	
**pTNM**			0.024			0.442			0.001
I	17 (81.0)	4 (19.0)		8 (38.1)	13 (61.9)		6 (28.6)	15 (71.4)	
II	30 (47.6)	33 (52.4)		15 (23.8)	48 (76.2)		47 (74.6)	16 (25.4)	
III	36 (50.7)	35 (49.3)		19 (26.8)	52 (73.2)		48 (67.6)	23 (32.4)	
**TB**			0.149			0.203			0.624
Bd1–2	65 (57.0)	49 (43.0)		34 (29.8)	80 (70.2)		73 (64.0)	41 (36.0)	
Bd3	18 (43.9)	23 (56.1)		8 (19.5)	33 (80.5)		28 (68.3)	13 (31.7)	
**Adjuvant chemotherapy**			0.198			0.317			0.149
No	42 (59.2)	29 (40.8)		22 (31.0)	49 (69.0)		42 (59.2)	29 (40.8)	
Yes	41 (48.8)	43 (51.2)		20 (23.8)	64 (76.2)		59 (70.2)	25 (29.8)	
**Sphincter-preserving**			0.919			0.116			0.198
No	12 (54.5)	10 (45.5)		9 (40.9)	13 (59.1)		17 (77.3)	5 (22.7)	
Yes	71 (53.4)	62 (46.6)		33 (24.8)	100 (75.2)		84 (63.2)	49 (36.8)	

a Pearson’s chi-squared test; Yates continuity correction chi-squared test.

### Prognostic significance of TLS maturation subtypes

Emerging evidence indicates that the density and maturation status of TLSs in the tumor immune microenvironment are closely associated with prognosis in various solid tumors [26]. However, the prognostic significance of the TLS maturation subtypes in pMMR RC remains poorly understood.

**Figure 3.**
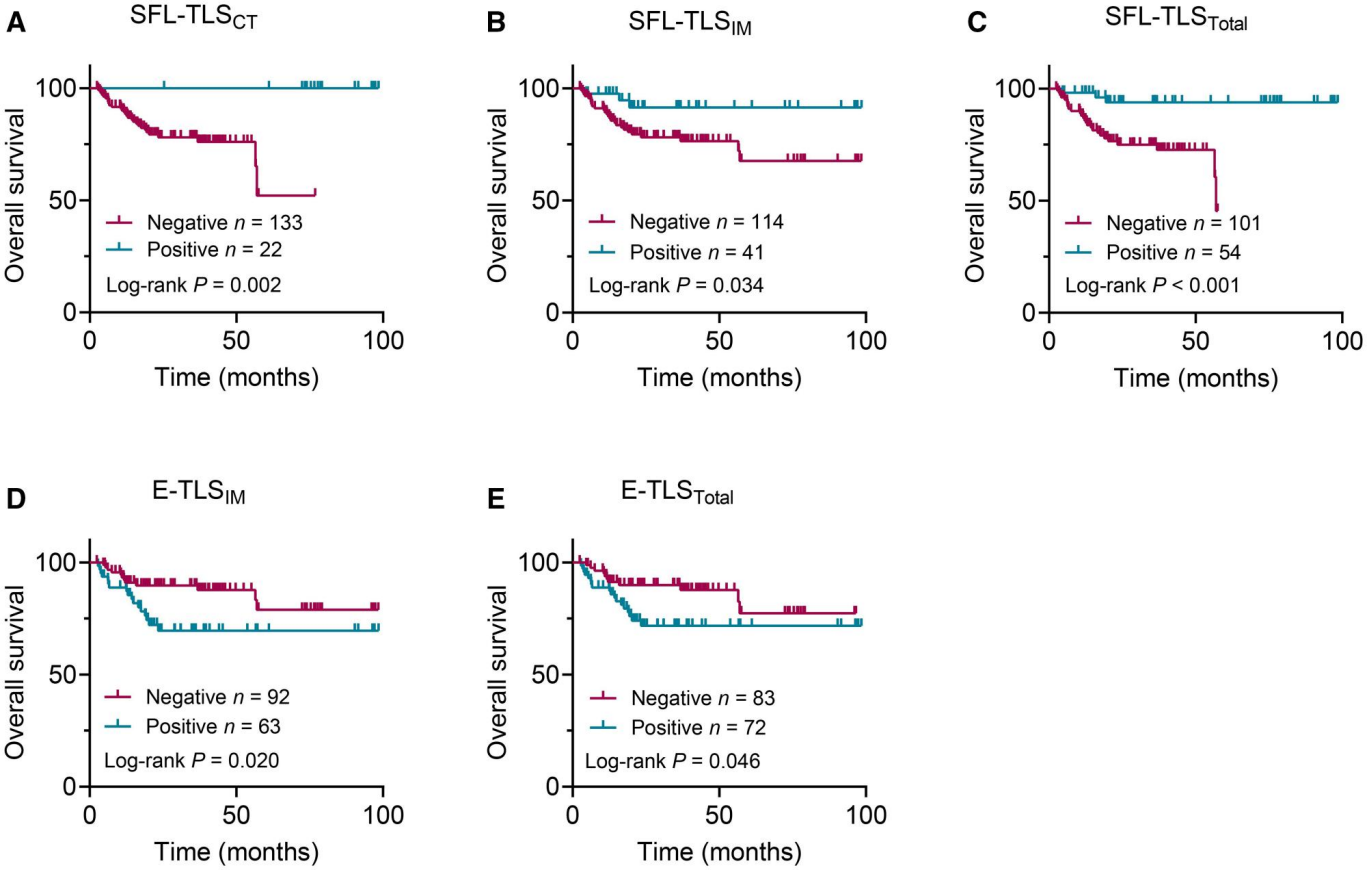
Assessment of the prognostic value of TLSs in pMMR RC. Kaplan–Meier curves illustrate the duration of OS associated with the infiltration status of (A) SFL-TLS_CT_, (B) SFL-TLS_IM_, (C) SFL-TLS_Total_, (D) E-TLS_IM_, and (E) E-TLS_Total_.

**Figure 4.**
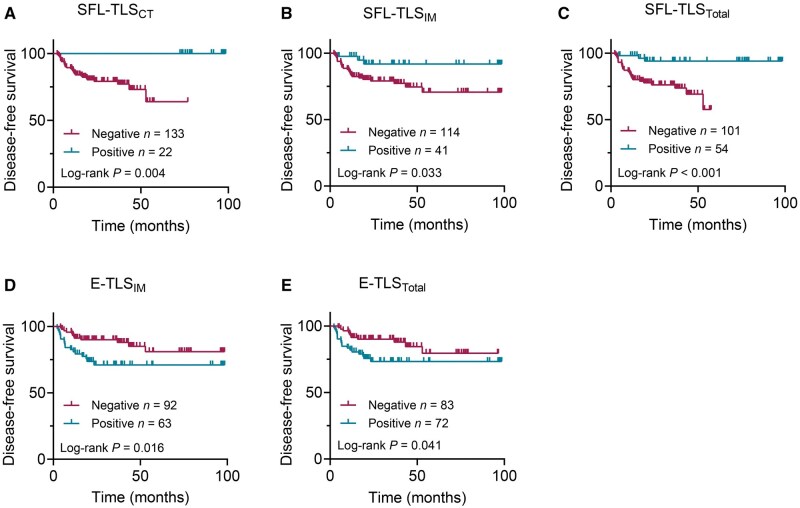
Assessment of the prognostic value of TLSs in pMMR RC. Kaplan–Meier curves illustrate the duration of DFS associated with the infiltration status of (A) SFL-TLS_CT_, (B) SFL-TLS_IM_, (C) SFL-TLS_Total_, (D) E-TLS_IM_, and (E) E-TLS_Total_..

We performed comprehensive association analyses between the infiltration status of TLS maturation subtypes, clinicopathological factors, and patient outcomes. Univariate survival analysis revealed that SFL-TLSs in any tumor region were positively associated with overall survival (OS) (Figure 3A–C) and disease-free survival (DFS) (Figure 4A–C). Conversely, the presence of E-TLSs in the IM region and overall tumor area was negatively associated with both OS (Figure 3D and E) and DFS (Figure 4D and E). Notably, the distribution of E-TLSs in the CT region showed no significant association with OS or DFS (Tables 3 and 4). Among clinicopathological factors, male gender, LVI, PNI, high-grade TB (Bd3), administration of adjuvant chemotherapy, and advanced pTNM stage were all negatively associated with OS (Table 3) and DFS (Table 4), indicating poorer survival among patients with these characteristics. Multivariate Cox regression analysis identified male gender (OS: hazard ratio [HR], 3.986; *P *= 0.005; DFS: HR, 5.080; *P *= 0.001), PNI (OS: HR, 7.490; *P *< 0.001; DFS: HR, 6.739; *P *< 0.001), and high-grade TB (OS: HR, 3.562; *P *= 0.001; DFS: HR, 3.186; *P *= 0.003) as independent risk factors for OS and DFS. Strikingly, the presence of SFL-TLSs in the overall tumor area emerged as an independent protective factor for OS (HR, 0.147; *P *= 0.002) and DFS (HR, 0.150; *P *= 0.002), indicating a significantly reduced mortality risk in patients with abundant SFL-TLS infiltration.

**Table 3.** Univariate and multivariate analysis of factors associated with OS for pMMR RC

Variable	Univariate analysis (log-rank test)		Multivariate analysis (Cox regression)		
	*χ* ^2^	*P*	HR	95% CI	*P*
Age (years)	0.227	0.634			
≤61					
>61					
Tumor size (cm)	0.735	0.391			
≤3.5					
>3.5					
Gender	6.880	0.009	3.986	1.505–10.555	0.005
Male					
Female					
LVI	4.845	0.028			
Negative					
Positive					
PNI	28.571	<0.001	7.490	3.128–17.933	<0.001
Negative					
Positive					
Tumor differentiation	0.287	0.592			
Poor/moderate					
Well					
pT	8.365	0.004			
pT1–2					
pT3–4					
pN	7.936	0.005			
pN0					
pN+					
pTNM	10.091	0.006			
I					
II					
III					
TB	9.482	0.002	3.562	1.683–7.538	0.001
Bd1–2					
Bd3					
Adjuvant chemotherapy	5.365	0.021			
No					
Yes					
Sphincter-preserving	0.3521	0.553			
No					
Yes					
E-TLS_CT_	0.147	0.702			
Negative					
Positive					
SFL-TLS_CT_	9.557	0.002			
Negative					
Positive					
E-TLS_IM_	5.380	0.020			
Negative					
Positive					
SFL-TLS_IM_	4.517	0.034			
Negative					
Positive					
E-TLS_Total_	3.975	0.046			
Negative					
Positive					
SFL-TLS_Total_	11.833	<0.001	0.147	0.043–0.499	0.002
Negative					
Positive					

CI = confidence interval.

**Table 4.** Univariate and multivariate analysis of factors associated with DFS for pMMR RC

Variable	Univariate analysis (log-rank test)		Multivariate analysis (Cox regression)		
	*χ* ^2^	*P*	HR	95% CI	*P*
Age (years)	0.220	0.639			
≤61					
>61					
Tumor size (cm)	0.718	0.397			
≤3.5					
>3.5					
Gender	7.566	0.006	5.080	1.876–13.751	0.001
Male					
Female					
LVI	4.136	0.042			
Negative					
Positive					
PNI	25.608	<0.001	6.739	2.738–16.585	<0.001
Negative					
Positive					
Tumor differentiation	0.298	0.585			
Poor/Moderate					
Well					
pT	7.901	0.005			
pT1–2					
pT3–4					
pN	8.135	0.004			
pN0					
pN+					
pTNM	10.175	0.006			
I					
II					
III					
TB	9.714	0.002	3.186	1.500–6.766	0.003
Bd1					
Bd2					
Bd3					
Adjuvant chemotherapy	5.403	0.020			
No					
Yes					
Sphincter-preserving	0.4041	0.525			
No					
Yes					
E-TLS_CT_	0.143	0.705			
Negative					
Positive					
SFL-TLS_CT_	8.216	0.004			
Negative					
Positive					
E-TLS_IM_	5.828	0.016			
Negative					
Positive					
SFL-TLS_IM_	4.561	0.033			
Negative					
Positive					
E-TLS_Total_	4.163	0.041			
Negative					
Positive					
SFL-TLS_Total_	11.109	<0.001	0.150	0.044–0.506	0.002
Negative					
Positive					

CI = confidence interval.

These findings identify SFL-TLS infiltration as a robust, independent prognostic biomarker for pMMR RC, highlighting its potential clinical utility in risk stratification and outcome prediction.

## Discussion

In this study, we systematically investigated the regional distribution and maturation subtypes of TLSs in pMMR RC. The principal findings can be summarized as follows: first, mature TLS subtypes were predominantly located in the IM; second, PFL-TLSs exhibited the highest infiltration levels across all tumor subregions; and, third, SFL-TLSs demonstrated a significant inverse association with advanced pT and pTNM stage, and were confirmed as an independent positive prognostic factor for both OS and DFS, whereas E-TLSs were linked to poorer outcomes.

Furthermore, our analysis provides deeper mechanistic and contextual insights into these observations. TLS maturation subtypes were predominantly located in the IM, consistently with reports indicating that TLS formation is more active in immunologically enriched peripheral regions [7, 27]. The IM region is characterized by a unique microenvironment enriched with immune cells, chemokines (e.g. CXCL13, CCL21), and inflammatory factors that collectively foster TLS formation and maturation [28, 29]. The enhanced antigen exposure and vascularization of this area facilitate immune cell recruitment and organization, establishing a permissive niche for TLS development [30]. Notably, the IM region also exhibits elevated expression of lymphoid-organizing molecules such as lymphotoxin-β and IL-7, further supporting its role as a hotspot for TLS initiation [29].

In addition, we identified notable functional distinctions among TLS subtypes. The widespread infiltration of PFL-TLSs across tumor subregions implies its potential role as a precursor to more mature TLS variants. As an early-stage TLS subtype, PFL-TLSs might initiate antitumor immunity by recruiting and activating immune cells, such as B cells and T cells, by secreting lymphoid-organizing chemokines and establishing immune cell niches [31, 32]. Recent studies have shown that PFL-TLSs can serve as a reservoir for tumor-reactive lymphocytes, potentially enhancing the efficacy of ICB therapies [33, 34]. These findings underscore the dynamic interplay between TLS spatial distribution, maturation, and the tumor immune microenvironment, offering insights into potential therapeutic strategies targeting TLS induction and maturation.

Moreover, we observed that SFL-TLSs were inversely associated with the pT stage and pTNM stage, suggesting that SFL-TLSs might suppress tumor progression. As a mature TLS subtype, SFL-TLSs enhance antitumor immune responses by promoting antigen presentation and T-cell activation [31, 35]. Importantly, univariate and multivariate survival analyses confirmed that SFL-TLS infiltration was positively associated with OS and DFS, whereas E-TLS infiltration was linked to poorer prognosis.

These results highlight the critical role of the TLS maturation status in determining patient outcomes. TLS maturation, particularly the transition from E-TLSs to SFL-TLSs, appears to be a critical determinant of antitumor efficacy. SFL-TLSs are characterized by the presence of a well-formed germinal center, which is a critical site for B-cell affinity maturation, class-switch recombination, and the generation of high-affinity, tumor-specific antibodies [7, 28]. This structured microenvironment not only supports effective B-cell responses, but also facilitates the activation and expansion of tumor-specific T cells through efficient antigen presentation by follicular dendritic cells and other antigen-presenting cells [36]. The coordinated interaction between B cells and T cells within these organized structures is believed to foster a potent and sustained antitumor immune response [29]. In contrast, due to their incomplete maturation, E-TLSs fail to effectively activate immune responses and could even promote tumor immune evasion by secreting immunosuppressive factors [19]. These findings emphasize the need for therapeutic strategies to promote TLS maturation to enhance antitumor immunity and improve patient outcomes.

Our findings are consistent with those of previous studies on the role of TLSs in the tumor immune microenvironment. For instance, multiple studies have shown that TLSs are associated with improved prognosis in various cancers, including melanoma, clear cell renal cell carcinoma, and bladder cancer [31–33]. In both melanoma and bladder cancer, the presence of TLSs was associated with improved patient outcomes and higher response rates to immunotherapy [37]. Our work focused on pMMR RC, systematically analysing the association between the maturity of TLSs and clinicopathological factors. Notably, we identified an inverse association between SFL-TLSs and tumor stage, providing new insights into the immune microenvironment of pMMR RC. Furthermore, our study showed that SFL-TLS infiltration was an independent protective factor for OS and DFS in pMMR RC, offering a potential biomarker for prognostic assessment. The novelty of our study lies in (i) it being a systematic characterization of TLS spatial distribution patterns in pMMR RC, (ii) the identification of functional differences among TLS subtypes across tumor regions, and (iii) the proposal that SFL-TLSs are a potential prognostic biomarker and novel target for immunotherapy in pMMR RC.

Despite these significant findings, our study had some limitations. First, as a retrospective study with a relatively small sample, our conclusions require validation through larger prospective studies. Second, our reliance on immunohistochemical techniques limited our ability to explore the molecular mechanisms underlying TLS formation and function. Future studies could integrate single-cell sequencing and spatial transcriptomics to further elucidate the cellular composition and functional states of TLSs. Additionally, our study did not address the relationship between TLSs and immunotherapy response. Future research should investigate the predictive value of TLSs in patients with pMMR RC undergoing ICB therapy.

In summary, we delineated the spatial distribution patterns of TLS maturation subtypes in pMMR RC and their clinical significance, particularly highlighting SFL-TLSs as an independent protective factor. These findings provide a foundation for TLS-centered immunotherapeutic development and prognostic stratification in pMMR RC.

## Supplementary Material

goag002_Supplementary_Data
