# Supplementary figures and images for: Secondary follicle-like TLS as a protective factor in pMMR rectal cancer: insights into its regional distribution and prognostic value
Source: Gastroenterol Rep (Oxf). 2026 Feb 13;14:goag002. doi: 10.1093/gastro/goag002 (PMC12902789; doi:10.1093/gastro/goag002)

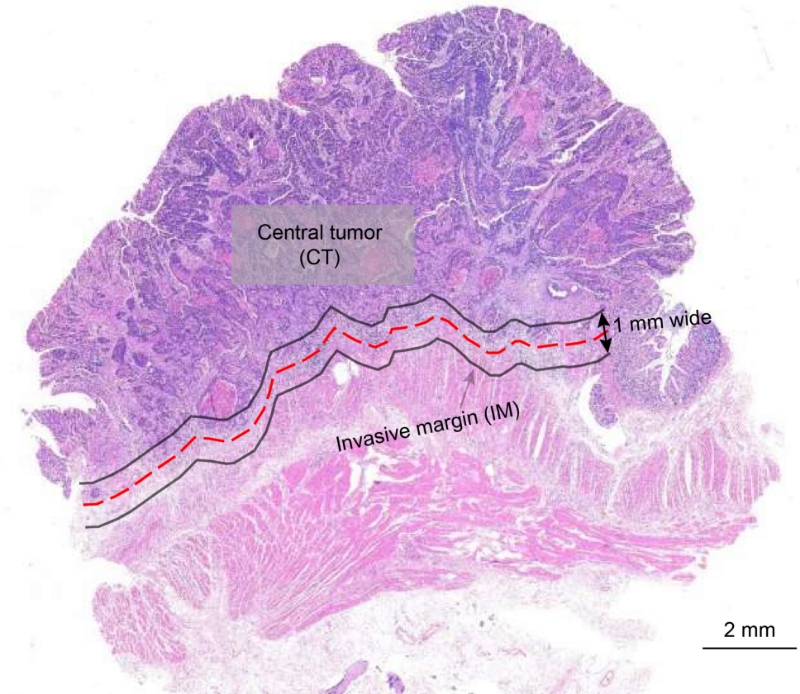

Supplement: goag002_Supplementary_Data [file goag002_supplementary_data.zip › 2025-204 Suppl Fig S1.tif]

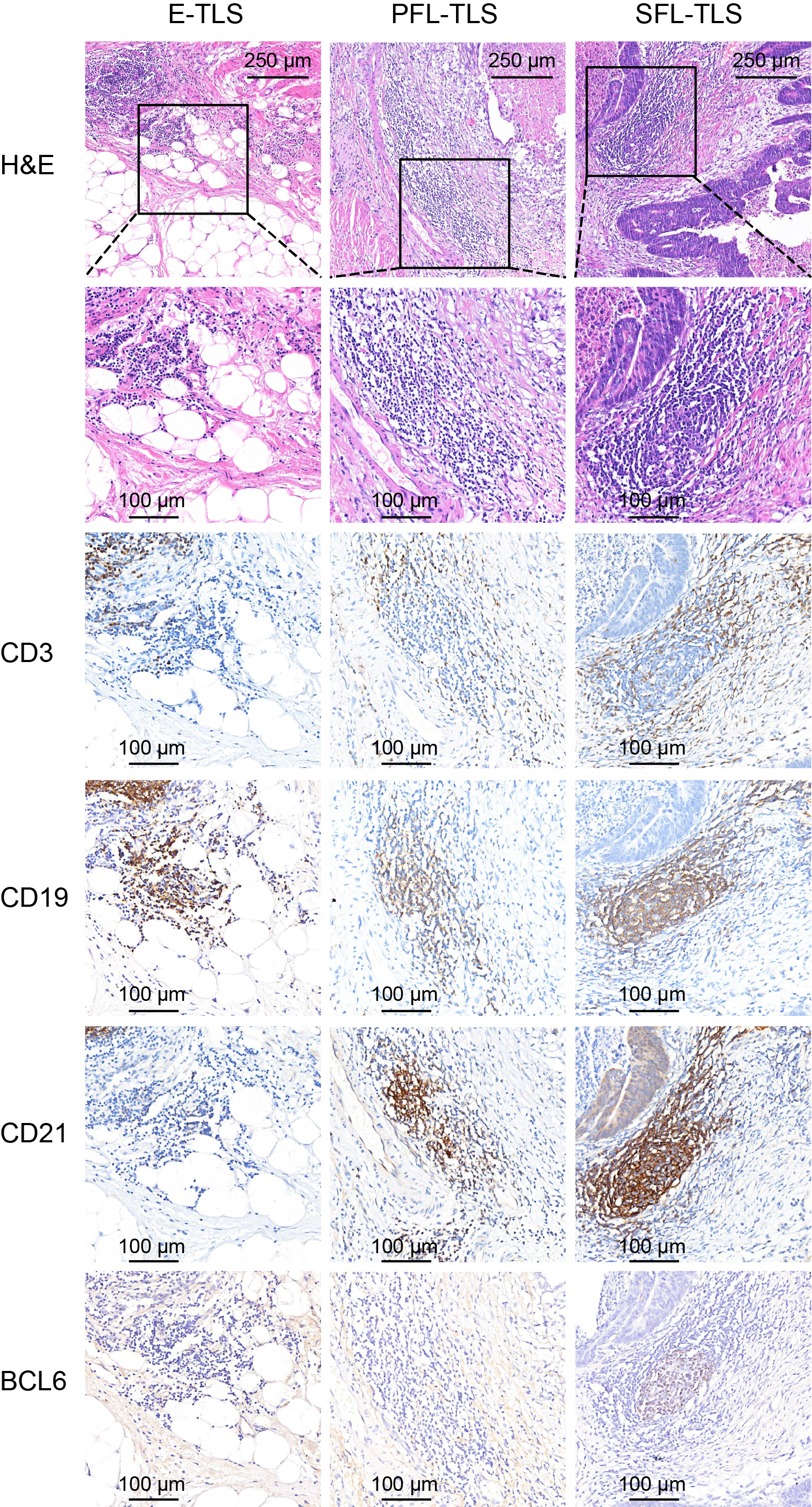

Supplement: goag002_Supplementary_Data [file goag002_supplementary_data.zip › 2025-204 Suppl Fig S2.tif]
